# Methyl 1-(7-acetamido-5,8-dimeth­oxy­quinolin-2-yl)-4-methyl-β-carboline-3-carboxyl­ate

**DOI:** 10.1107/S1600536811018794

**Published:** 2011-05-25

**Authors:** Felix Nissen, Dieter Schollmeyer, Heiner Detert

**Affiliations:** a University Mainz, Duesbergweg 10-14, 55099 Mainz, Germany

## Abstract

The title compound, C_27_H_24_N_4_O_5_, is an inter­mediate in the synthesis of lavendamycin *via* a ruthenium-catalysed [2 + 2 + 2] cyclo­addition. An intra­molecular hydrogen-bond bridge from the carboline to the quinoline stabilizes a highly planar geometry [maximum deviation = 0.065 (6) Å] for the two rigid units. This hydrogen-bond-stabilized coplanarity has a very close analogy in the structure of the anti­tumor anti­biotic streptonigrin in the solid state and in solution. Inter­molecular hydrogen-bond bridges of amides groups along the *a* axis and π–π stacking inter­actions [centroid–centroid distance = 3.665 (9) Å] connect mol­ecules arranged in a parallel manner.

## Related literature

For metal-catalysed transformations of tethered alkynyl-ynamides to carbolines and other heteroannulated indoles, see: Nissen *et al.* (2011 ▶); Dassonneville *et al.* (2010 ▶, 2011 ▶). For the synthesis of the natural product lavendamycin (systematic name 1-(7-amino-5,8-dioxoquinolin-2-yl)-4-methyl-9*H*-pyrido[3,4-*b*]indole-3-carb­oxy­lic acid) *via* [2 + 2 + 2] cyclo­addition, see: Nissen & Detert (2011 ▶). For the isololation of lavendamycin from *streptomyces lavendulae*, see: Doyle *et al.* (1981 ▶). For the anti-tumor activity of lavendamycin, see: Fang *et al.* (2003 ▶). For the preparation of lavandamycin, see: Behforouz *et al.* (1996 ▶) Godard *et al.* (1993 ▶). For related structures, see: Chiu & Lipscomb (1975 ▶); Harding *et al.* (1993 ▶).
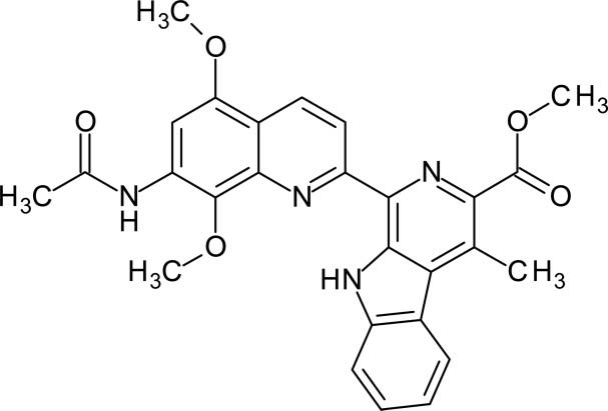


## Experimental

### 

#### Crystal data

C_27_H_24_N_4_O_5_
*M* *_r_* = 484.50
Triclinic, 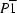

*a* = 4.646 (10) Å
*b* = 13.81 (3) Å
*c* = 18.768 (19) Å
α = 102.06 (9)°
β = 95.23 (10)°
γ = 96.85 (11)°
*V* = 1161 (4) Å^3^
*Z* = 2
Cu *K*α radiation
μ = 0.80 mm^−1^
*T* = 193 K
0.58 × 0.06 × 0.03 mm

#### Data collection

Enraf–Nonius CAD-4 diffractometer
4993 measured reflections
4412 independent reflections
2069 reflections with *I* > 2σ(*I*)
*R*_int_ = 0.054
3 standard reflections every 60 min  intensity decay: 2%

#### Refinement

*R*[*F* ^2^ > 2σ(*F* ^2^)] = 0.083
*wR*(*F* ^2^) = 0.248
*S* = 0.99
4412 reflections
330 parameters
H-atom parameters constrained
Δρ_max_ = 0.26 e Å^−3^
Δρ_min_ = −0.34 e Å^−3^

### 

Data collection: *CAD-4 Software* (Enraf–Nonius, 1989 ▶); cell refinement: *CAD-4 Software*; data reduction: *CORINC* (Dräger & Gattow, 1971 ▶); program(s) used to solve structure: *SIR97* (Altomare *et al.*, 1999 ▶); program(s) used to refine structure: *SHELXL97* (Sheldrick, 2008 ▶); molecular graphics: *PLATON* (Spek, 2009 ▶); software used to prepare material for publication: *PLATON*.

## Supplementary Material

Crystal structure: contains datablocks I, global. DOI: 10.1107/S1600536811018794/bt5551sup1.cif

Structure factors: contains datablocks I. DOI: 10.1107/S1600536811018794/bt5551Isup2.hkl

Supplementary material file. DOI: 10.1107/S1600536811018794/bt5551Isup3.cml

Additional supplementary materials:  crystallographic information; 3D view; checkCIF report

## Figures and Tables

**Table 1:** Hydrogen-bond geometry (Å, °)

*D*—H⋯*A*	*D*—H	H⋯*A*	*D*⋯*A*	*D*—H⋯*A*
N2—H2⋯N3	0.88	2.17	2.693 (7)	118
N4—H4⋯O5^i^	0.88	1.98	2.824 (8)	160

Symmetry code: (i) 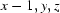.

**Fig. 1.**
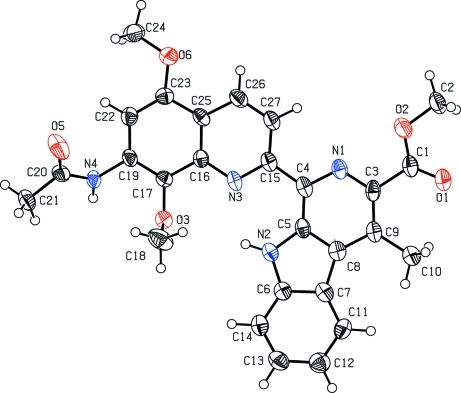
View of compound I. Displacement ellipsoids are drawn at the 50% probability level.

**Fig. 2.**
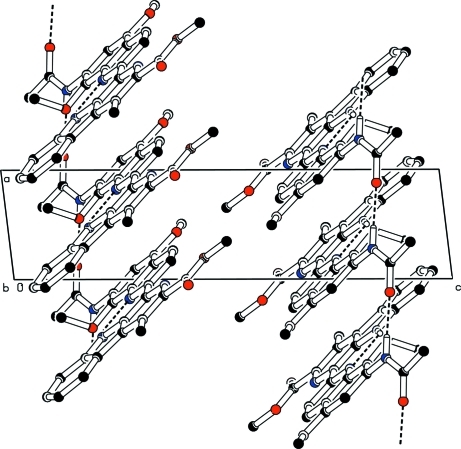
Part of the packing diagram showing the hydrogen bonds. View along b-axis.

## supplementary crystallographic information

### Comment

The title compound was prepared as part of a larger project focusing on the synthesis of indolo-annulated heterocycles *via* [2 + 2+2] cycloaddition of alkynyl-ynamides and nitriles or heterocumulenes catalyzed by rhodium or ruthenium (Nissen *et al.*, 2011; Dassonneville *et al.*, 2011) and is a synthetic precursor of the natural product lavendamycin (Nissen & Detert, 2011). The molecule is built up by two rigid and planar units, both are coplanar. This flat structure is stabilized by an intramolecular hydrogen bond from the carboline-NH to the quinoline-N N2—H2···N3 with a distance of 2.17 Å. Whereas the ester group is coplanar with the carboline core [torsion angle O1—C1—C3—C9 of -0.4 (8)°], the acetamido group is twisted out of the plane of the quinoline framework. The torsion angle C20—N4—C19—C22 amounts to -47.9 (7)°. This torsion can result from the sterical hindrance due to the neighbouring methoxy group, but its ability to act as a hydrogen bridge donor and acceptor is important for the formation of crystals of the title compound. Parallel molecules, arranged in the shape of tilted staples are connected to infinite chains *via* H-bridging along the *a*-axis. The tilt angle of the mean plane of the molecules relative to the *ab*-plane is 54.3°. The second interaction is π-π-stacking, resulting in a distance of only 3.665 (9) Å of the centroids of the pyrrole ring (N2—C6) and the benzo-ring (C6—C14)  (symmetry code 1+*X*,Y,*Z*). Two molecules, connected *via* a center of inversion fill the unit cell.

### Experimental

Pd/C (10%, 47 mg, 44 µmol, 5 mol%) was added to a suspension of Methyl 1-(5,8-dimethoxy-7-nitroquinolin-2-yl)-4-methyl-β-carboline-3-carboxylate (405 mg, 0.86 mmol) (Nissen & Detert, 2011) in THF (170 ml) and the mixture was stirred 13 h under H_2_ atmosphere. Ac_2_O (5.0 ml) was added and the mixture was heated to 323 K. After 3 h the solvent was removed *in vacuo*, the residue dissolved in CH_2_Cl_2_ and filtered through celite. The filtrate was washed with aqueous NaHCO_3_ (8%, 8 ml), dried (MgSO_4_) and concentrated. Crystallization from chloroform/diethyl ether yielded the title compound (399 mg, 0.82 mmol, 96%) as a bright yellow solid. *M*.p. 467–468 K *R*_f_: 0.12 (SiO_2_, hexane:ethyl acetate:ethanol 6:3:1).

### Refinement

Hydrogen atoms attached to carbons were placed at calculated positions with C—H = 0.95 Å (aromatic) or 0.98–0.99 Å (*sp*^3^ C-atom). All H atoms were refined in the riding-model approximation with isotropic displacement parameters set at 1.2–1.5 times of the *U*_eq_ of the parent atom.

### Crystal data

**Table:**

C_27_H_24_N_4_O_5_	*Z* = 2
*M**_r_* = 484.50	*F*(000) = 508
Triclinic, *P*1	*D*_x_ = 1.386 Mg m^−^^3^
Hall symbol: -P 1	Cu *K*α radiation, λ = 1.54178 Å
*a* = 4.646 (10) Å	Cell parameters from 25 reflections
*b* = 13.81 (3) Å	θ = 10–25°
*c* = 18.768 (19) Å	µ = 0.80 mm^−^^1^
α = 102.06 (9)°	*T* = 193 K
β = 95.23 (10)°	10, yellow
γ = 96.85 (11)°	0.58 × 0.06 × 0.03 mm
*V* = 1161 (4) Å^3^	

### Data collection

**Table:**

Enraf–Nonius CAD-4 diffractometer	*R*_int_ = 0.054
Radiation source: rotating anode	θ_max_ = 70.0°, θ_min_ = 2.4°
graphite	*h* = −5→0
ω/2θ scans	*k* = −16→16
4993 measured reflections	*l* = −22→22
4412 independent reflections	3 standard reflections every 60 min
2069 reflections with *I* > 2σ(*I*)	intensity decay: 2%

### Refinement

**Table:**

Refinement on *F*^2^	Secondary atom site location: difference Fourier map
Least-squares matrix: full	Hydrogen site location: inferred from neighbouring sites
*R*[*F*^2^ > 2σ(*F*^2^)] = 0.083	H-atom parameters constrained
*wR*(*F*^2^) = 0.248	*w* = 1/[σ^2^(*F*_o_^2^) + (0.1159*P*)^2^] where *P* = (*F*_o_^2^ + 2*F*_c_^2^)/3
*S* = 0.99	(Δ/σ)_max_ < 0.001
4412 reflections	Δρ_max_ = 0.26 e Å^−^^3^
330 parameters	Δρ_min_ = −0.34 e Å^−^^3^
0 restraints	Extinction correction: *SHELXL97* (Sheldrick, 2008), Fc^*^=kFc[1+0.001xFc^2^λ^3^/sin(2θ)]^-1/4^
Primary atom site location: structure-invariant direct methods	Extinction coefficient: 0.0010 (5)

### Special details

**Table:**

Geometry. All e.s.d.'s (except the e.s.d. in the dihedral angle between two l.s. planes) are estimated using the full covariance matrix. The cell e.s.d.'s are taken into account individually in the estimation of e.s.d.'s in distances, angles and torsion angles; correlations between e.s.d.'s in cell parameters are only used when they are defined by crystal symmetry. An approximate (isotropic) treatment of cell e.s.d.'s is used for estimating e.s.d.'s involving l.s. planes.
Refinement. Refinement of *F*^2^ against ALL reflections. The weighted *R*-factor *wR* and goodness of fit *S* are based on *F*^2^, conventional *R*-factors *R* are based on *F*, with *F* set to zero for negative *F*^2^. The threshold expression of *F*^2^ > σ(*F*^2^) is used only for calculating *R*-factors(gt) *etc*. and is not relevant to the choice of reflections for refinement. *R*-factors based on *F*^2^ are statistically about twice as large as those based on *F*, and *R*- factors based on ALL data will be even larger.

### Fractional atomic coordinates and isotropic or equivalent isotropic displacement parameters (Å^2^)

**Table:**

	*x*	*y*	*z*	*U*_iso_*/*U*_eq_	
O1	0.9484 (9)	0.6415 (3)	0.3958 (2)	0.0631 (12)	
O2	1.1937 (9)	0.5349 (3)	0.43953 (19)	0.0530 (11)	
O3	0.5569 (7)	−0.0529 (2)	0.16500 (18)	0.0381 (8)	
N4	0.7247 (8)	−0.2335 (3)	0.1735 (2)	0.0314 (9)	
H4	0.5329	−0.2404	0.1660	0.038*	
O5	1.1210 (8)	−0.3053 (3)	0.1464 (2)	0.0691 (14)	
O6	1.4309 (8)	−0.0606 (3)	0.38367 (19)	0.0469 (10)	
N1	0.9463 (9)	0.3816 (3)	0.3424 (2)	0.0363 (10)	
N2	0.4724 (9)	0.2191 (3)	0.1858 (2)	0.0367 (10)	
H2	0.4994	0.1561	0.1806	0.044*	
N3	0.8225 (9)	0.1206 (3)	0.2585 (2)	0.0349 (10)	
C1	0.9992 (12)	0.5579 (4)	0.3929 (3)	0.0392 (12)	
C2	1.3460 (13)	0.6178 (4)	0.4953 (3)	0.0506 (15)	
H2A	1.4680	0.6626	0.4725	0.076*	
H2B	1.4695	0.5926	0.5305	0.076*	
H2C	1.2039	0.6545	0.5210	0.076*	
C3	0.8488 (11)	0.4675 (3)	0.3360 (3)	0.0362 (12)	
C4	0.8328 (11)	0.2958 (3)	0.2955 (3)	0.0361 (12)	
C5	0.6143 (11)	0.2973 (3)	0.2397 (3)	0.0341 (11)	
C6	0.2800 (11)	0.2556 (4)	0.1412 (3)	0.0352 (11)	
C7	0.2906 (11)	0.3594 (4)	0.1682 (3)	0.0355 (11)	
C8	0.5118 (11)	0.3860 (3)	0.2323 (3)	0.0344 (11)	
C9	0.6334 (11)	0.4754 (3)	0.2817 (3)	0.0368 (12)	
C10	0.5268 (13)	0.5721 (4)	0.2747 (3)	0.0508 (15)	
H10A	0.4642	0.6039	0.3214	0.076*	
H10B	0.3616	0.5585	0.2359	0.076*	
H10C	0.6850	0.6169	0.2623	0.076*	
C11	0.1095 (11)	0.4101 (4)	0.1309 (3)	0.0416 (13)	
H11	0.1105	0.4800	0.1476	0.050*	
C12	−0.0723 (12)	0.3573 (4)	0.0691 (3)	0.0490 (14)	
H12	−0.1993	0.3914	0.0439	0.059*	
C13	−0.0731 (12)	0.2558 (4)	0.0431 (3)	0.0477 (14)	
H13	−0.1969	0.2220	−0.0002	0.057*	
C14	0.0991 (12)	0.2038 (4)	0.0782 (3)	0.0432 (13)	
H14	0.0965	0.1340	0.0605	0.052*	
C15	0.9462 (11)	0.2043 (3)	0.3056 (3)	0.0367 (12)	
C16	0.9155 (11)	0.0334 (3)	0.2670 (3)	0.0320 (11)	
C17	0.7812 (10)	−0.0564 (3)	0.2176 (3)	0.0330 (11)	
C18	0.6517 (15)	−0.0421 (5)	0.0971 (3)	0.0645 (19)	
H18A	0.7902	0.0195	0.1047	0.097*	
H18B	0.4831	−0.0388	0.0627	0.097*	
H18C	0.7473	−0.0996	0.0769	0.097*	
C19	0.8703 (10)	−0.1450 (3)	0.2232 (3)	0.0323 (11)	
C20	0.8550 (10)	−0.3067 (4)	0.1373 (3)	0.0374 (12)	
C21	0.6628 (11)	−0.3906 (4)	0.0857 (3)	0.0447 (14)	
H21A	0.7272	−0.3984	0.0368	0.067*	
H21B	0.4610	−0.3762	0.0833	0.067*	
H21C	0.6731	−0.4525	0.1029	0.067*	
C22	1.0881 (11)	−0.1513 (4)	0.2785 (3)	0.0373 (12)	
H22	1.1437	−0.2143	0.2820	0.045*	
C23	1.2197 (10)	−0.0657 (4)	0.3273 (3)	0.0338 (11)	
C24	1.5056 (13)	−0.1523 (4)	0.3972 (3)	0.0514 (15)	
H24A	1.3311	−0.1930	0.4064	0.077*	
H24B	1.6528	−0.1387	0.4403	0.077*	
H24C	1.5846	−0.1885	0.3544	0.077*	
C25	1.1358 (11)	0.0293 (3)	0.3224 (3)	0.0345 (11)	
C26	1.2625 (12)	0.1205 (4)	0.3705 (3)	0.0413 (13)	
H26	1.4150	0.1214	0.4080	0.050*	
C27	1.1656 (11)	0.2061 (4)	0.3626 (3)	0.0392 (12)	
H27	1.2453	0.2676	0.3955	0.047*	

### Atomic displacement parameters (Å^2^)

**Table:**

	*U*^11^	*U*^22^	*U*^33^	*U*^12^	*U*^13^	*U*^23^
O1	0.068 (3)	0.032 (2)	0.077 (3)	0.006 (2)	−0.016 (2)	−0.004 (2)
O2	0.073 (3)	0.037 (2)	0.042 (2)	−0.003 (2)	−0.006 (2)	0.0036 (17)
O3	0.038 (2)	0.0337 (18)	0.0398 (19)	0.0018 (16)	−0.0003 (17)	0.0052 (15)
N4	0.022 (2)	0.0242 (19)	0.044 (2)	0.0010 (16)	0.0027 (18)	0.0013 (17)
O5	0.0185 (19)	0.067 (3)	0.097 (3)	0.0068 (18)	−0.001 (2)	−0.033 (2)
O6	0.047 (2)	0.039 (2)	0.050 (2)	0.0009 (17)	−0.0097 (18)	0.0100 (17)
N1	0.039 (2)	0.028 (2)	0.040 (2)	−0.0042 (18)	0.009 (2)	0.0057 (18)
N2	0.036 (2)	0.031 (2)	0.038 (2)	−0.0019 (18)	0.002 (2)	0.0024 (18)
N3	0.038 (2)	0.027 (2)	0.034 (2)	−0.0025 (18)	0.0078 (19)	−0.0016 (17)
C1	0.042 (3)	0.029 (3)	0.044 (3)	−0.002 (2)	0.012 (3)	0.003 (2)
C2	0.069 (4)	0.033 (3)	0.041 (3)	−0.006 (3)	0.000 (3)	0.000 (2)
C3	0.047 (3)	0.024 (2)	0.038 (3)	0.001 (2)	0.016 (2)	0.007 (2)
C4	0.040 (3)	0.032 (3)	0.033 (3)	−0.004 (2)	0.013 (2)	0.001 (2)
C5	0.033 (3)	0.027 (2)	0.039 (3)	−0.002 (2)	0.010 (2)	0.003 (2)
C6	0.031 (3)	0.037 (3)	0.037 (3)	0.004 (2)	0.008 (2)	0.005 (2)
C7	0.030 (3)	0.039 (3)	0.035 (3)	0.001 (2)	0.011 (2)	0.002 (2)
C8	0.034 (3)	0.036 (3)	0.034 (3)	0.001 (2)	0.015 (2)	0.008 (2)
C9	0.042 (3)	0.028 (2)	0.038 (3)	0.000 (2)	0.012 (2)	0.002 (2)
C10	0.058 (4)	0.038 (3)	0.050 (3)	0.004 (3)	−0.007 (3)	0.004 (3)
C11	0.041 (3)	0.035 (3)	0.052 (3)	0.010 (2)	0.010 (3)	0.011 (3)
C12	0.041 (3)	0.052 (3)	0.053 (3)	0.004 (3)	0.004 (3)	0.012 (3)
C13	0.039 (3)	0.050 (3)	0.048 (3)	−0.002 (3)	−0.002 (3)	0.004 (3)
C14	0.045 (3)	0.035 (3)	0.047 (3)	0.001 (2)	0.007 (3)	0.005 (2)
C15	0.040 (3)	0.031 (3)	0.036 (3)	−0.002 (2)	0.006 (2)	0.002 (2)
C16	0.033 (3)	0.028 (2)	0.034 (3)	0.001 (2)	0.005 (2)	0.006 (2)
C17	0.029 (3)	0.033 (3)	0.034 (3)	0.002 (2)	0.000 (2)	0.005 (2)
C18	0.075 (5)	0.071 (4)	0.040 (3)	−0.016 (4)	−0.004 (3)	0.016 (3)
C19	0.028 (3)	0.025 (2)	0.039 (3)	−0.003 (2)	0.001 (2)	0.002 (2)
C20	0.020 (2)	0.040 (3)	0.046 (3)	0.004 (2)	−0.003 (2)	−0.001 (2)
C21	0.032 (3)	0.039 (3)	0.054 (3)	0.002 (2)	0.002 (3)	−0.009 (3)
C22	0.038 (3)	0.032 (3)	0.041 (3)	0.003 (2)	0.005 (2)	0.008 (2)
C23	0.026 (2)	0.038 (3)	0.037 (3)	0.001 (2)	0.003 (2)	0.011 (2)
C24	0.047 (3)	0.049 (3)	0.057 (4)	0.003 (3)	−0.008 (3)	0.020 (3)
C25	0.032 (3)	0.034 (3)	0.033 (3)	−0.006 (2)	0.002 (2)	0.003 (2)
C26	0.040 (3)	0.038 (3)	0.038 (3)	−0.007 (2)	−0.004 (2)	0.004 (2)
C27	0.043 (3)	0.031 (3)	0.037 (3)	−0.003 (2)	0.002 (2)	0.001 (2)

### Geometric parameters (Å, °)

**Table:**

O1—C1	1.198 (6)	C16—C17	1.420 (7)
O2—C1	1.313 (6)	C17—C19	1.359 (7)
O2—C2	1.442 (6)	C19—C22	1.406 (7)
O3—C17	1.380 (6)	C20—C21	1.485 (7)
O3—C18	1.417 (7)	C22—C23	1.373 (7)
N4—C20	1.336 (6)	C23—C25	1.431 (7)
N4—C19	1.425 (6)	C25—C26	1.414 (7)
O5—C20	1.229 (6)	C26—C27	1.346 (7)
O6—C23	1.360 (6)	N2—H2	0.8800
O6—C24	1.419 (6)	N4—H4	0.8800
N1—C4	1.337 (6)	C2—H2A	0.9800
N1—C3	1.342 (6)	C2—H2B	0.9800
N2—C5	1.370 (6)	C2—H2C	0.9800
N2—C6	1.380 (6)	C10—H10A	0.9800
N3—C15	1.330 (6)	C10—H10B	0.9800
N3—C16	1.362 (6)	C10—H10C	0.9800
C1—C3	1.512 (7)	C11—H11	0.9500
C3—C9	1.392 (7)	C12—H12	0.9500
C4—C5	1.396 (7)	C13—H13	0.9500
C4—C15	1.467 (7)	C14—H14	0.9500
C5—C8	1.395 (7)	C18—H18A	0.9800
C6—C14	1.392 (7)	C18—H18B	0.9800
C6—C7	1.410 (7)	C18—H18C	0.9800
C7—C11	1.388 (7)	C21—H21A	0.9800
C7—C8	1.462 (7)	C21—H21B	0.9800
C8—C9	1.400 (7)	C21—H21C	0.9800
C9—C10	1.505 (7)	C22—H22	0.9500
C11—C12	1.383 (7)	C24—H24A	0.9800
C12—C13	1.383 (8)	C24—H24B	0.9800
C13—C14	1.352 (7)	C24—H24C	0.9800
C15—C27	1.402 (7)	C26—H26	0.9500
C16—C25	1.406 (7)	C27—H27	0.9500
			
C1—O2—C2	115.5 (4)	C16—C25—C26	117.4 (5)
C17—O3—C18	113.6 (4)	C16—C25—C23	119.0 (4)
C20—N4—C19	125.5 (4)	C26—C25—C23	123.6 (5)
C23—O6—C24	117.3 (4)	C27—C26—C25	119.5 (5)
C4—N1—C3	120.2 (5)	C26—C27—C15	119.7 (5)
C5—N2—C6	108.6 (4)	C6—N2—H2	126.00
C15—N3—C16	117.6 (4)	C5—N2—H2	126.00
O1—C1—O2	123.1 (5)	C19—N4—H4	117.00
O1—C1—C3	124.4 (5)	C20—N4—H4	117.00
O2—C1—C3	112.5 (4)	O2—C2—H2A	110.00
N1—C3—C9	124.4 (4)	O2—C2—H2B	109.00
N1—C3—C1	113.9 (5)	O2—C2—H2C	109.00
C9—C3—C1	121.7 (4)	H2A—C2—H2B	109.00
N1—C4—C5	118.9 (5)	H2A—C2—H2C	109.00
N1—C4—C15	117.8 (5)	H2B—C2—H2C	109.00
C5—C4—C15	123.2 (4)	C9—C10—H10A	109.00
N2—C5—C8	110.2 (4)	C9—C10—H10B	109.00
N2—C5—C4	128.5 (5)	C9—C10—H10C	110.00
C8—C5—C4	121.4 (4)	H10A—C10—H10B	109.00
N2—C6—C14	128.4 (5)	H10A—C10—H10C	109.00
N2—C6—C7	109.4 (4)	H10B—C10—H10C	109.00
C14—C6—C7	122.2 (5)	C7—C11—H11	120.00
C11—C7—C6	118.2 (5)	C12—C11—H11	121.00
C11—C7—C8	136.1 (5)	C11—C12—H12	119.00
C6—C7—C8	105.7 (4)	C13—C12—H12	119.00
C5—C8—C9	119.0 (5)	C12—C13—H13	119.00
C5—C8—C7	106.1 (4)	C14—C13—H13	119.00
C9—C8—C7	134.8 (5)	C6—C14—H14	121.00
C3—C9—C8	116.0 (5)	C13—C14—H14	121.00
C3—C9—C10	124.0 (4)	O3—C18—H18A	110.00
C8—C9—C10	120.0 (5)	O3—C18—H18B	109.00
C12—C11—C7	119.0 (5)	O3—C18—H18C	109.00
C11—C12—C13	121.4 (5)	H18A—C18—H18B	109.00
C14—C13—C12	121.3 (5)	H18A—C18—H18C	109.00
C13—C14—C6	118.0 (5)	H18B—C18—H18C	109.00
N3—C15—C27	123.1 (5)	C20—C21—H21A	109.00
N3—C15—C4	115.5 (5)	C20—C21—H21B	109.00
C27—C15—C4	121.4 (4)	C20—C21—H21C	110.00
N3—C16—C25	122.7 (4)	H21A—C21—H21B	109.00
N3—C16—C17	117.9 (4)	H21A—C21—H21C	109.00
C25—C16—C17	119.4 (4)	H21B—C21—H21C	109.00
C19—C17—O3	120.5 (4)	C19—C22—H22	120.00
C19—C17—C16	120.0 (4)	C23—C22—H22	120.00
O3—C17—C16	119.5 (4)	O6—C24—H24A	109.00
C17—C19—C22	121.7 (4)	O6—C24—H24B	109.00
C17—C19—N4	118.0 (4)	O6—C24—H24C	109.00
C22—C19—N4	120.2 (4)	H24A—C24—H24B	109.00
O5—C20—N4	121.8 (5)	H24A—C24—H24C	109.00
O5—C20—C21	121.4 (5)	H24B—C24—H24C	110.00
N4—C20—C21	116.8 (4)	C25—C26—H26	120.00
C23—C22—C19	119.5 (5)	C27—C26—H26	120.00
O6—C23—C22	125.9 (5)	C15—C27—H27	120.00
O6—C23—C25	113.7 (4)	C26—C27—H27	120.00
C22—C23—C25	120.5 (5)		
			
C2—O2—C1—O1	−0.2 (8)	N2—C6—C14—C13	179.9 (5)
C2—O2—C1—C3	179.9 (4)	C7—C6—C14—C13	−0.7 (8)
C4—N1—C3—C9	−1.0 (7)	C16—N3—C15—C27	0.1 (7)
C4—N1—C3—C1	−179.9 (4)	C16—N3—C15—C4	−178.3 (4)
O1—C1—C3—N1	178.5 (5)	N1—C4—C15—N3	178.7 (4)
O2—C1—C3—N1	−1.6 (6)	C5—C4—C15—N3	−1.4 (7)
O1—C1—C3—C9	−0.4 (8)	N1—C4—C15—C27	0.3 (7)
O2—C1—C3—C9	179.5 (5)	C5—C4—C15—C27	−179.8 (5)
C3—N1—C4—C5	0.6 (7)	C15—N3—C16—C25	−0.6 (7)
C3—N1—C4—C15	−179.5 (4)	C15—N3—C16—C17	179.1 (5)
C6—N2—C5—C8	1.5 (5)	C18—O3—C17—C19	−88.9 (6)
C6—N2—C5—C4	−178.0 (5)	C18—O3—C17—C16	92.3 (6)
N1—C4—C5—N2	179.1 (5)	N3—C16—C17—C19	179.0 (4)
C15—C4—C5—N2	−0.8 (8)	C25—C16—C17—C19	−1.4 (7)
N1—C4—C5—C8	−0.4 (7)	N3—C16—C17—O3	−2.3 (7)
C15—C4—C5—C8	179.7 (4)	C25—C16—C17—O3	177.4 (4)
C5—N2—C6—C14	177.4 (5)	O3—C17—C19—C22	−176.8 (4)
C5—N2—C6—C7	−2.0 (5)	C16—C17—C19—C22	2.0 (7)
N2—C6—C7—C11	−179.4 (4)	O3—C17—C19—N4	−0.2 (7)
C14—C6—C7—C11	1.1 (7)	C16—C17—C19—N4	178.6 (4)
N2—C6—C7—C8	1.7 (5)	C20—N4—C19—C17	135.4 (5)
C14—C6—C7—C8	−177.8 (5)	C20—N4—C19—C22	−47.9 (7)
N2—C5—C8—C9	−178.9 (4)	C19—N4—C20—O5	2.7 (8)
C4—C5—C8—C9	0.6 (7)	C19—N4—C20—C21	−177.5 (4)
N2—C5—C8—C7	−0.4 (5)	C17—C19—C22—C23	−1.4 (7)
C4—C5—C8—C7	179.1 (4)	N4—C19—C22—C23	−178.0 (5)
C11—C7—C8—C5	−179.3 (6)	C24—O6—C23—C22	−5.6 (7)
C6—C7—C8—C5	−0.8 (5)	C24—O6—C23—C25	173.7 (4)
C11—C7—C8—C9	−1.1 (10)	C19—C22—C23—O6	179.5 (5)
C6—C7—C8—C9	177.4 (5)	C19—C22—C23—C25	0.2 (7)
N1—C3—C9—C8	1.2 (7)	N3—C16—C25—C26	−0.1 (7)
C1—C3—C9—C8	179.9 (5)	C17—C16—C25—C26	−179.8 (5)
N1—C3—C9—C10	−179.7 (5)	N3—C16—C25—C23	179.9 (4)
C1—C3—C9—C10	−0.9 (8)	C17—C16—C25—C23	0.2 (7)
C5—C8—C9—C3	−1.0 (7)	O6—C23—C25—C16	−179.0 (4)
C7—C8—C9—C3	−179.0 (5)	C22—C23—C25—C16	0.3 (7)
C5—C8—C9—C10	179.9 (5)	O6—C23—C25—C26	1.0 (7)
C7—C8—C9—C10	1.9 (8)	C22—C23—C25—C26	−179.7 (5)
C6—C7—C11—C12	−0.1 (7)	C16—C25—C26—C27	1.4 (7)
C8—C7—C11—C12	178.3 (5)	C23—C25—C26—C27	−178.7 (5)
C7—C11—C12—C13	−1.2 (8)	C25—C26—C27—C15	−1.8 (8)
C11—C12—C13—C14	1.6 (9)	N3—C15—C27—C26	1.1 (8)
C12—C13—C14—C6	−0.6 (8)	C4—C15—C27—C26	179.5 (5)

### Hydrogen-bond geometry (Å, °)

**Table:**

*D*—H···*A*	*D*—H	H···*A*	*D*···*A*	*D*—H···*A*
N2—H2···N3	0.88	2.17	2.693 (7)	118
N4—H4···O5^i^	0.88	1.98	2.824 (8)	160

Symmetry codes: (i) *x*−1, *y*, *z*.
